# Ribosome heterogeneity and specialization

**DOI:** 10.1098/rstb.2023.0375

**Published:** 2025-03-06

**Authors:** Julie Aspden, William James Faller, Maria Barna, Anders Lund

**Affiliations:** ^1^ School of Molecular and Cellular Biology, Faculty of Biological Sciences, University of Leeds, Leeds LS2 9JT, UK; ^2^ LeedsOmics, University of Leeds, Leeds LS2 9JT, UK; ^3^ Astbury Centre for Structural Molecular Biology, University of Leeds, Leeds, LS2 9JT, UK; ^4^ Netherlands Cancer Institute, Amsterdam, The Netherlands; ^5^ Stanford University, Stanford, CA, USA; ^6^ Biotech Research and Innovation Centre, University of Copenhagen, Kobenhavn DK-2200, Denmark

**Keywords:** mRNA translation, ribosomal proteins, protein synthesis, ribosome, rRNA modifications

## 1. Historical perspective

The historical journey of ribosome research has undergone significant shifts, evolving from an initial perception of ribosomes as uniform molecular machines to their current recognition as dynamic regulators of translation. In the mid-1950s, George Palade, using electron microscopy, first described ribosomes as small particulate structures in the cytoplasm, which he initially termed ‘microsomes’ [1]. These structures were later identified as sites of protein synthesis, and the term ‘ribosome’ was officially coined at a 1958 Biophysical Society symposium. Shortly after their discovery, the notion of ribosome heterogeneity was introduced by Francis Crick in his ‘one gene–one ribosome–one protein’ hypothesis [2], which proposed that each ribosome was specialized to synthesize a single protein. However, this hypothesis was quickly challenged in the early 1960s by experiments from Sydney Brenner, François Jacob and Matthew Meselson, whose research facilitated the notion that ribosomes are non-specialized structures that synthesize different proteins depending on the mRNA they contain [3]. These findings led to the prevailing dogma that ribosomes function as passive, homogeneous entities in translation, a perspective that dominated molecular biology for decades.

Despite this early dismissal, sporadic observations of ribosome heterogeneity emerged throughout the late twentieth century [4]. Studies in the 1980s and 1990s identified tissue-specific ribosomal protein (RP) paralogue expression in plants and invertebrates, as well as variation in ribosomal RNA (rRNA) sequences in *Plasmodium* [5] during different life cycle stages. In the 1990s and at the turn of the century, researchers discovered that certain RPs were essential for specific developmental processes in *Drosophila* [6] and vertebrates [7], leading to distinct phenotypic effects rather than global translation defects (reviewed in [8]). By the early 2000s, advances in molecular biology techniques, including ribosome profiling and quantitative mass spectrometry, began revealing direct evidence of ribosome specialization. For instance, studies demonstrated that actively translating ribosomes in mouse embryonic stem cells exhibited compositional diversity [9], with specific ribosomal proteins influencing the translation of distinct subsets of mRNAs [10]. Ribosome heterogeneity now extends to stress states in bacteria [11] and yeast [12,13], neurons [14], germ line [15,16], development [17,18] and immunity [19],

to name a few.

Thereby, the story of ribosome research is not just one of scientific discovery but also of shifting paradigms in how we understand biological complexity. At its core, it is a reflection of a broader philosophical tension in biology: the struggle between reductionism and emergent complexity. The early view of ribosomes as uniform molecular machines aligned with the reductionist framework that dominated mid-twentieth century molecular biology—a time when the central dogma of gene expression was being solidified. In this model, the ribosome was merely a passive conduit for genetic information, faithfully translating mRNA into protein without bias or specialization. This perspective offered a comforting simplicity, reducing the complexity of life to a set of uniform, predictable biochemical processes. However, as history has shown time and again, nature resists oversimplification. The emerging evidence of ribosome heterogeneity challenges this mechanistic view, forcing a shift toward a more dynamic, systems-level understanding of translation and gene regulation. This further favours the notion that, whenever in evolution you can regulate, you do, to achieve greater and greater complexity to life.

In many ways, the ribosome embodies the philosophical concept of plasticity—its form and function are not fixed but instead adapt to the physiological and developmental needs of the organism. This mirrors the broader theme in biology that identity is not static but context-dependent, a principle seen in stem cell differentiation, neural plasticity and even ecological adaptation. The discovery that ribosomes are specialized, that they selectively translate different mRNAs in different tissues and developmental stages, suggests that life is not merely governed by a universal set of molecular rules but is instead sculpted by intricate regulatory networks that respond to the needs of the organism. This recognition moves us away from the deterministic idea that genes alone dictate biological outcomes, and toward a more holistic view in which the cell, the tissue and even the ribosome contribute to the final expression of genetic potential. Ribosome specialization, then, is not just a molecular mechanism—it is a reflection of life’s ability to fine-tune itself, to carve out specificity and meaning from what was once thought to be uniform and universal. This is reflected by the fact that today’s researchers are actively exploring how ribosomal composition influences gene regulation, cellular differentiation and disease states, positioning ribosome heterogeneity as a fundamental mechanism in our understanding of the central dogma and the complexity and plasticity of life as we know it.

## 2. Royal Society meeting

In November 2023, a Hooke Discussion Meeting brought together international scientists to share perspectives and novel insights on ribosome heterogeneity and specialization. This meeting discussed the latest advances, covering all types of ribosome heterogeneity in a variety of organisms and systems, including impact on human disease. The meeting was organized by Dr Julie Aspden, Dr Maria Barna, Dr William Faller and Dr Anders H. Lund. Contributions came from numerous disciplines, such as structural biology, cancer biology, biochemistry, developmental biology and neuroscience. Talks and posters focused on a range of different types of ribosome heterogeneity, including ribosome protein paralogue switching events, rRNA modification changes during development and cancer, and association of modulatory proteins to the core ribosome.

Importantly, the community is attempting to unravel the functional implications of ribosome heterogeneity using a wide variety of systems and approaches. The latest results come from *Drosophila melanogaster*, *Danio rerio*, human intestinal and embryonic stem cells, viral infection, yeast and plants. The study of different examples in different species will be essential in understanding how widespread different types of specialization are and how similar translational impacts can be achieved through different changes in ribosome composition. A good example of this is the variety of ways the peptide exit channel can be modified to enable the regulation of nascent peptide folding and elongation dynamics [16].

Talks and posters covered the biogenesis of heterogeneous ribosomes, what stimulates changes in ribosome composition (e.g. stresses), how changes affect the structure of the ribosome, the impact of these changes on translation and how this impacts the biological function of the cells, tissues and organism, including disease phenotypes.

## 3. Technical advances

A common theme among the talks was the advances in technology that are driving the field forward. As with all young fields, there have been teething problems with the technologies used to identify and analyse specialized ribosomes. By their nature, ribosomes are difficult to study, and sequencing, proteomic and visualization techniques have all presented problems. However, as the field has developed, each has been adapted to this purpose and the toolbox available is growing steadily.

Adaptations of high-throughput approaches have proven particularly fruitful. While sequencing technologies have been used for over a decade to identify modifications of rRNA [20,21], the development of long-read sequencing, and associated analysis tools, has provided a new window into this [22,23]. Similar approaches have also been adapted to allow the analysis of rRNA transcript variation. The high number and homology of rDNA genes have previously prevented the reliable analysis of different rRNA transcripts, something that has been overcome using long-read sequencing [24].

Approaches to study the protein complement of the ribosome have also been adapted to handle the difficulties that come with studying RPs and ribosome-associated proteins (RAPs). In the coming years, expansion microscopy should allow the direct *in vivo* visualization of ribosome populations, although this is yet to happen. The recently described targeted RNase H-mediated extraction of cross(X)-linked ribosomal binding proteins (TREX) methodology provides an improved means to identify proteins directly binding to the rRNA [25], while both the RAP identification by affinity to sulfhydryl-charged resin (RAPIDASH) [26] and RNA affinity purification using poly-lysine (RAPPL) [27] approaches describe improvements to the isolation of ribosomes and their associated proteins. RAPPL-isolated ribosomes have been visualized at high resolution using cryogenic electron microscopy (cryoEM), a technology that itself has seen significant recent improvements [28–31]. Specialized ribosomes have only been directly visualized a limited number of times [15,16], and these advances, along with those in cryogenic electron tomography (cryoET) [32], highlight the potential of these technologies to shape our understanding of ribosome biology.

## 4. Outstanding questions

While there have been important conceptual and technological advances in the field, many central questions remain unanswered.

First, how are specialized ribosomes generated from the arrays of rDNA and how are these processes controlled in the cell? In extension, how is the composition of specialized ribosomes regulated during normal processes, such as cellular differentiation, and how are they hijacked in diseases?

Second, whereas heterologous ribosome populations have been described from all branches of life, how can we identify and characterize distinct ribosome subtypes with potentially different specialization modalities? Here, single molecule sequencing of rRNA modification patterns could be an important stepping stone [33]. Evidently, improved ribosome tagging or purification methods will also need to be developed to unravel this puzzle.

Third, how is ribosome specialization organized in the cell? While we know of several examples of ‘translation factories’, unique cellular locations where specific proteins are synthesized [34], it is currently not known how such factories are generated, and—importantly—how specific mRNAs are transported to factories or specialized ribosomes.

Fourth, given the evolutionary conservation and high number of ribosomes in the cells, will we see additional functions not directly linked to protein synthesis [35]?

Finally, in many tumour types, the ribosome composition and modification patterns are altered—presumably to facilitate elevated protein synthesis and potentially deviant translation patterns. If such ‘oncoribosomes’ can be specifically targeted, it could curtail many cancers with an ‘addiction’ to elevated protein synthesis.

This special issue contains research articles, reviews and perspectives covering the key themes discussed at the Hooke Meeting (November 2023). Several discuss the potential of employing cutting-edge approaches to study ribosome heterogeneity and specialization and look to the future of the field. We hope you enjoy this collection of articles on ribosome heterogeneity and specialization in this special issue.

## Data Availability

This article has no additional data.
